# TRP Ion Channels in Immune Cells and Their Implications for Inflammation

**DOI:** 10.3390/ijms25052719

**Published:** 2024-02-27

**Authors:** Qiyue Yan, Chuanzhou Gao, Mei Li, Rui Lan, Shaohan Wei, Runsong Fan, Wei Cheng

**Affiliations:** Institute of Cancer Stem Cell, Dalian Medical University, Dalian 116044, China; qiyueyan0000@gmail.com (Q.Y.); gaocz@dmu.edu.cn (C.G.); limei791@dmu.edu.cn (M.L.); lanrui699@gmail.com (R.L.); weishaohan666@gmail.com (S.W.); runsongf@gmail.com (R.F.)

**Keywords:** TRP ion channel, immune cells, pro-inflammation, anti-inflammation, cytokine

## Abstract

The transient receptor potential (TRP) ion channels act as cellular sensors and mediate a plethora of physiological processes, including somatosensation, proliferation, apoptosis, and metabolism. Under specific conditions, certain TRP channels are involved in inflammation and immune responses. Thus, focusing on the role of TRPs in immune system cells may contribute to resolving inflammation. In this review, we discuss the distribution of five subfamilies of mammalian TRP ion channels in immune system cells and how these ion channels function in inflammatory mechanisms. This review provides an overview of the current understanding of TRP ion channels in mediating inflammation and may offer potential avenues for therapeutic intervention.

## 1. Introduction

The inflammatory response represents the physiological defense mechanism against infection and injury, facilitating the elimination and removal of injurious factors and damaged tissue components to initiate the healing process [1,2]. Acute inflammation refers to a transient response lasting only a few days, whereas chronic inflammation denotes a prolonged reaction. A prominent characteristic of inflammation is the accumulation and/or infiltration of immune cells (macrophages, lymphocytes, and plasma cells) at the site of injury, recruited from the circulation system through the continuous release of chemotactic factors by damaged tissues [3,4,5,6]. Pro-inflammatory factors encompass viruses, bacteria, physical trauma, injury, chemical agents, inappropriate immunological responses, and tissue necrosis. Additionally, ischemia caused by oxygen or nutrient deprivation can induce an inflammatory state [4,7,8,9].

The mammalian TRP ion channels comprise 28 members that are distributed among six subfamilies, namely, TRPA (ankyrin), TRPC (canonical), TRPM (melastatin), TRPV (vanilloid), TRPML (mucolipin), and TRPP (polycystin) [10,11]. The majority of these TRPs exhibit permeability to non-selective cations. Functioning as molecular sensors, they play a crucial role in various physiological and pathological processes. These versatile TRP ion channels respond to stimuli such as temperature, pH, pressure, chemicals, noxious agents, and microorganisms that can trigger inflammation [12,13,14,15,16]. Notably present in immune cells and tissues, the regulation of expression, gating mechanisms, and subsequent modulation of ion homeostasis by these ion channels significantly impact inflammatory responses [17,18]. The present review primarily focuses on the involvement of TRP ion channels in immune system cells while discussing their implications as mediators in both pro-inflammatory and anti-inflammatory responses.

## 2. Distribution of TRP Ion Channels in Immune Cells

The immune system cells are divided into innate leukocytes and adaptive lymphocytes, both derived from hematopoietic stem cells in the bone marrow. Innate leukocytes contain phagocytes (neutrophils, macrophages, and dendritic cells (DCs)), mast cells, eosinophils, basophils, and natural killer (NK) cells. Adaptive lymphocytes harbor B and T cells. Notably, TRP ion channels have been reported to be expressed in nearly all immune system cell types to date.

### 2.1. TRPs in Macrophages

When inflammation is initiated, a large number of immune cells migrate to the site of injury, including neutrophils, and monocytes, which eventually differentiate into phagocytic macrophages. Macrophages typically become more abundant at the injury site only after a span of days or weeks, thus serving as a cellular hallmark indicative of chronic inflammation. In response to microenvironmental cues, macrophages can transform from primitive cells to either M1 (pro-inflammatory) or M2 (anti-inflammatory) phenotypes. Upon lipopolysaccharide (LPS) stimulation, M2 macrophages have elevated expression of TRPC1 that supersedes that of Orai1 in these cells [19]. TRPC3 has been identified as a novel effector for LPS-triggered Ca^2+^ signaling in primitive macrophages [20]. One study indicates that the expression of TRPC5 negatively regulates the transformation of macrophages into the M1 phenotype by inhibiting their polarization [21]. In the human and rat osteoarthritis synovium, TRPV1 tends to be expressed in M1 phenotypes rather than M2 [22]. Sanjai Kumar et al. reported that Chikungunya virus (CHIKV), a virus that induces a pathogenic inflammatory host immune response, up-regulated the expression of TRPV1 in macrophages [23]. Upon application of LPS as well as SARS-CoV-2 infection, established macrophage cell lines and mouse bone marrow-derived macrophages polarize towards M1 with expression of TRPV2 [24,25]. TRPV4 has been confirmed to be abundantly present in rat alveolar macrophages (AMs) through LPS stimulation [26]. TRPM2 is expressed in mouse macrophages [27,28,29]. While peritoneal and bone marrow-derived macrophages also exhibit essential expression of TRPM4 [30]. Additionally, various approaches have revealed the presence of the TRPM7 in murine primary splenocytes, naive CD4^+^ T cells, peritoneal mast cells, and macrophages, suggesting its significance in monocyte-derived cells [31]. Magnesium imaging techniques have demonstrated the induction of currents upon extracellular addition of Mg^2+^, indicating the expression of TRPM7 in human monocyte-derived macrophages as well as mouse bone marrow-derived macrophages [32,33]. Furthermore, both the RAW 264.7 cell line and murine peritoneal macrophages exhibit significant enrichment of TRPM8 proteins [34]. Extracted from another source, it has been observed that primitive AMs obtained from consecutive lung lavages promote the expression of TRPML2 upon exposure to LPS. Interestingly, agonists of TRPML2 seem to ineffectively activate TRPML2 without LPS treatment, revealing the limited intrinsic expression of TRPML2 in macrophages [35]. Previous studies have demonstrated that cannabichromene (CBC) can induce activation of its membrane receptors, namely, TRPV1 and TRPA1, in mouse peritoneal macrophages [36,37].

Microglia, as a resident macrophage population within the central nervous system (CNS), play a crucial role in neurodevelopment and surveillance of CNS homeostasis [38]. Primary microglial cells isolated from the whole brain of 3-day-old postnatal Sprague Dawley rats exhibited robust TRPC3 immunoreactivity on their surface, which was further enhanced by brain-derived neurotrophic factor (BDNF) [39]. Similar to macrophages, upon stimulation of LPS, there was subsequent expression and activation of TRPV1 in mouse microglia [40]. Akyuva et al. demonstrated that homogeneous microglia residing in the brain of type C57BL/6J black mice displayed excessive currents of TRPM2 when stimulated with interferon-γ (IFN-γ) [41]. Furthermore, in murine BV2 microglial cells, SUR1-TRPM4 heteromers were formed through the assembly of TRPM4 with sulfonylurea receptor 1 (SUR1) [42].

### 2.2. TRPs in Mast Cells

Mast cells, derived from CD34^+^ hematopoietic stem cells, exhibit distinct phenotypes based on their localization in connective tissue and mucosal regions. To cope with the intricate milieu, especially in intestinal disorders, mast cells coordinate various cytokines involved in both the innate and adaptive immune responses [43,44]. Notably, it has been confirmed that TRPM4 induces a time-dependent current in bone marrow-derived mast cells; however, this current diminishes when TRPM4 is suppressed [45]. Furthermore, human mast cells possess cation selectivity and divalent permeability attributed to the presence of TRPM7 ion channel when intracellular Mg^2+^ levels are limited [46].

### 2.3. TRPs in Neutrophils

When confronted with external jeopardy, pathogen-relevant molecular patterns and/or damage-associated molecular patterns (DAMPs) bind to pattern recognition receptors as well as DAMP receptors, leading to an enhanced production of inflammatory mediators [47]. In 129Sv/C57BL/6J WT mice, TRPC1 ion channels are ubiquitously detected in the membrane of neutrophils. By utilizing Trpc1 knockout mice (Trpc1^−/−^), we can effectively investigate the role of TRPC1 in neutrophils [48]. Additionally, the deficiency of Trpc6 in 129Sv/C57BL/6J WT mice results in a loss of Ca^2+^ signaling in neutrophils [49]. Moreover, Wu et al. have demonstrated the presence of TRPV4 on the plasma membrane of neutrophils [50]. Furthermore, it has been shown that TRPM2 is sensitive to reactive oxygen species (ROS) during trans-endothelial processes in neutrophils [51]. CD16^+^ neutrophils, isolated from the whole blood of human donors, exhibit magnesium and Mg-ATP currents, indicating activation of TRPM7 ion channels in these cells [52].

### 2.4. TRPs in Specific Immunocytes

Dendritic cells (DCs) play a crucial role in initiating adaptive immune responses through pattern recognition receptors. Upon internalization of antigens via non-specific micropinocytosis and subsequent absorption into the cytoplasm, organelles commence antigen processing to generate small peptides with appropriate sizes and sequences [53]. Sumoza-Toledo et al. have reported the expression of TRPM2 mRNA in bone marrow-derived immature DCs [54]. Additionally, within DC subsets, approximately 10% of CD11c^−^ F480^+^, CD11c^+^ F480^−^, and non-T non-B cell populations consist of TRPV1^+^ cells [55,56]. Furthermore, Bretou et al. have provided precise localization information on TRPML1 within the lysosome of DCs [57].

The external stimulation triggers the maturation of DCs, leading to a reduction in antigen uptake and an up-regulation of CCR7 surface expression. This chemokine receptor recognizes gradients of CCL21 and CCL19, guiding DCs towards lymphatic vessels and lymph nodes where they present major histocompatibility complex (MHC) peptides to CD4^+^ and CD8^+^ T cells for immune response against invading microbes and aberrant host cells [58,59]. Following antigen presentation, cytokine signals coordinate and determine the lineage fate of activated CD4^+^ T cells, converting them into distinct subsets known as T-helper (Th) cells. To date, various Th cell subsets have been identified, including Th1, Th2, regulatory T (Treg), follicular helper T (Tfh), Th9, Th17, and Th22 cells [60]. Notably, TRP channels have been implicated in regulating inflammatory processes involving Th cell responses. A recent study suggests that the expression of TRPA1 and TRPV1 in neurons potentiates the recruitment of Th2 cells [61]. TRPC6 expression ensures Th2 functionality in the context of airway allergy [62]. Similarly, the colonization of Th17 cells with TRPM7 and its kinase expression triggers protection against acute graft-versus-host disease [31]. Additionally, TRPM7 involvement is attributed to the thymic development of Treg cells in T-cell-driven hepatitis [63]. A plethora of antigens endow CD8^+^ T cells with energetic and biosynthetic capacity, which fuels memory CD8^+^ T cell differentiation and exploits the synergy of long-term tissue immunity [64]. The negative regulatory role of TRPC3 expression on T cell activation, particularly CD8^+^ T cells, has been validated [65]. Furthermore, Acharya et al. have observed the endogenous signal of TRPV4 in resting murine T cells, excluding active ones from their study scope [66].

In the presence of a T-cell-dependent humoral immune response or antigen presented by DCs, B cells initiate a peripheral reaction and migrate to secondary lymphoid tissues. Regardless of the immune response from other immunotypes, the generation of memory B cells facilitates an adaptive immunity that is more durable and associated with the immunizing antigen. Furthermore, upon invasion of antigens, memory B cells exhibit enhanced proliferation and differentiation kinetics [67]. Sakaguchi et al. observed distinct levels of TRPM5 abundance in immature and mature B cells within the spleen rather than in the bone marrow [68]. Additionally, TRPML1 expression has been detected in NK cells purified from human peripheral blood mononuclear cells [69] (Table 1).

## 3. Involvement of TRPs in Inflammation

During the inflammatory response, a diverse array of cytokines is secreted by various immune cells to stimulate, recruit, and amplify the immune response. These cytokines can be broadly classified into two categories: pro-inflammatory and anti-inflammatory. Pro-inflammatory cytokines, such as interleukin (IL)-1, IL-6, IL-17, interferon (IFN)-γ, and tumor necrosis factor (TNF)-α, are primarily produced by Th1 cells, CD4^+^ T cells, macrophages, and DCs. Among them, IL-1, IL-6, and TNF-α cytokines play pivotal roles in regulating immune cell growth, activation, differentiation, and homing. Ultimately, these pro-inflammatory cytokines contribute to the effective control and elimination of intracellular pathogens, including viruses. IL-10, IL-12, and transforming growth factor (TGF)-β subtypes are released by various immune cells, such as T cells, B cells, macrophages, monocytes, and NK cells, to exert their anti-inflammatory effects [70,71,72]. Chemokines represent a group of cytokines with chemotactic activity that can be divided into four main subtypes: CXC, CC, CX3C, and XC. These chemokines play crucial roles in regulating the migration and localization of lymphocytes and DCs. Specifically, CXC chemokines primarily participate in the recruitment of immune cells to sites of inflammation. However, it is worth noting that certain chemokines exhibit dual functions, whereby their pro-inflammatory or anti-inflammatory properties depend on their distribution and concentration within the system [73,74,75].

### 3.1. Pro-inflammatory Effects of TRPs

**Figure 1:**
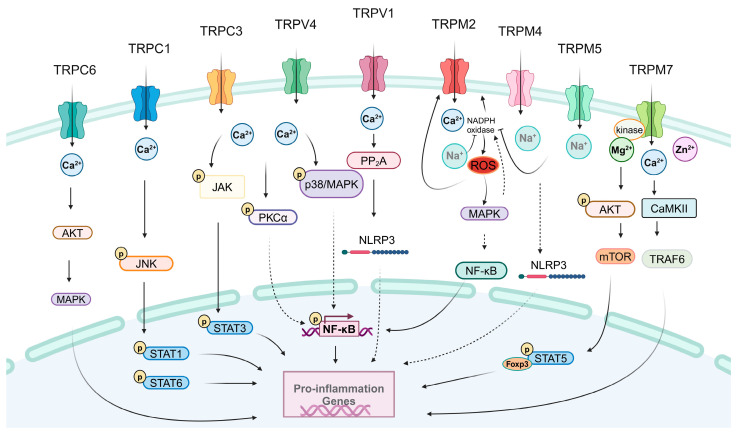
TRPs modulate inflammation in immune cells. The activation of TRPC6, TRPC3, TRPV4, and TRPM2 leads to Ca^2+^ influx, which then triggers the AKT/MAPK signaling pathway to enhance the expression of pro-inflammatory genes. When TRPC1 or TRPC3 is activated, it allows for increased calcium influx and subsequently activates the phosphorylation of JNK/STAT1 or STAT6, or JAK/STAT3, respectively, to promote the release of pro-inflammatory factors. Stimulation of TRPC3 induces pro-inflammatory gene expression via PKC signaling. Activation of the TRPV1 channel results in calcium entry and subsequent activation of PP2A to activate the NLRP3 inflammasome, thereby promoting an inflammatory response. When Ca^2+^ ions flow out of the lysosome via the TRPM2 ion channel, it leads to an increase in intracellular calcium concentration, which further activates the MAPK pathway. Inhibition of NADPH oxidase activity can enhance the production of reactive oxygen species (ROS) and activate the MAPK pathway, resulting in the phosphorylation of NF-κB and its translocation into the nucleus, thereby leading to the production of pro-inflammatory factors. Activation of TRPM4 and TRPM5 channels can induce sodium influx and trigger NLRP3 inflammasome activation, thus contributing to inflammation. Activation of TRPM7 triggers signaling pathways such as AKT/mTOR, STAT5-Foxp3, or Ca^2+^/CaMKII/TRAF6, leading to pro-inflammatory gene transcription.

Stimulation of TRP ion channels in immune system cells has been found to induce an increase in inflammatory cytokines, providing compelling evidence that the activation or inhibition of TRPs leads to a cascade of inflammatory responses with pronounced pro-inflammatory effects (Figure 1). Nascimento Da Conceicao et al. have found that Trpc1^−/−^ mice exhibit transcriptional changes that suppress the activities of pro-inflammatory cytokines. Blockage of Ca^2+^ entry attenuates pNF-κB/pJNK/STAT1 or STAT6 signaling, thereby subduing cytokine production [19]. LPS is present in endoplasmic reticulum (ER) membranes, where lipin-1 hydrolyzes phosphatidic acid (PA) pools sequentially to evoke macrophages to generate diacylglycerol (DAG), which further activates TRPC3 ion channels to ensure intracellular Ca^2+^ elevation. Ultimately, this triggers a cascade of cellular signaling that induces NF-κB translocation to the nucleus and up-regulation of inflammatory genes [20]. Jing et al. identified that overexpression of TRPC3 inhibits T cell activation, particularly CD8^+^ T lymphocytes, thus promoting inflammation-induced preterm labor [65]. TRPC6 is involved in the recruitment of neutrophils during inflammation. Loss of TRPC6 leads to a deficiency in CXCR2-mediated chemotaxis due to reduced intracellular Ca^2+^ levels in Trpc6^−/−^ neutrophils, resulting in impaired phosphorylation of AKT and MAPK downstream of the CXCR2 receptor, eventually hindering neutrophil recruitment [49]. In contrast to wide-type (WT) mice, Trpc6^−/−^ mice exhibit significantly diminished allergic responses following allergen challenge, including decreased blood IgE levels, reduced airway eosinophilia, and the absence of Th2 cytokines IL-5 and IL-13 in bronchoalveolar lavage fluid [62]. Furthermore, exposure to O_3_ stimulation results in impaired recruitment of neutrophils, macrophages, and lymphocytes into the airway as well as reduced production of the inflammatory factors IL-6, IL-8, and TNF-α in the bronchoalveolar lavage fluid of Trpc6^−/−^ mice or mice pretreated with the TRPC6 inhibitor SAR7334 [76].

The activation of TRPA1 initiates the release of ROS and the pro-inflammatory cytokines IL-1β and IL-6 by orchestrating the NF-κB signaling pathway in microglia [77]. Additionally, TRPA1 is involved in facilitating the release of the IL-8 cytokine and regulating the expression of matrix metalloprotease 9 (MMP9) in human lung fibroblasts, which are susceptible to TNF-α. Overall, these findings suggest that inflammatory mediators such as TNF-α have synergistic effects on TRPA1 in fibroblasts, potentially exacerbating the inflammatory cascade observed in human airway diseases [78].

The NLRP3 inflammasome plays a crucial role in the regulation of immunity and inflammation. Zhang et al. have validated that TRPV1 acts as a pivotal factor in the activation of the NLRP3 inflammasome [40]. Furthermore, modulation of TRPV1 by its antagonist also elicits an anti-inflammatory response. Specifically, certain TRPV1 antagonists, such as AMG9810 and capsaizepine, restrict the production of IL-6, IL-1β, IL-18, and cyclooxygenase-2 (COX-2) in murine macrophages [79]. Additionally, TRPV2 participates in manipulating the NF-κB-dependent TNF-α and IL-6 signal conversion by mediating intracellular Ca^2+^ mobilization. Specifically, extracellular Ca^2+^ entry promotes IL-6 production independently of NF-κB signaling pathway activation [80]. By abolishing Ca^2+^ entry through the TRPV4 channel, protease-activated receptor 2 conjugates the phosphorylated protease of AMs, sustains thrombin efflux to assemble the cAMP, and then invalidates TLR4 inflammatory signaling in AMs [26]. Dutta et al. conducted an analysis of emerging data and inferred that TRPV4 is involved in the remodeling of the extracellular matrix, leading to increased internal stiffness during inflammation. This process subsequently promotes macrophage polarization towards M1 inflammatory status [81]. Wu et al. demonstrated that TRPV4 agonists induce Ca^2+^ influx and facilitate ROS augmentation in neutrophils. The significant alteration in neutrophil infiltration and ROS production exacerbates myocardial ischemia/reperfusion injury [50]. Similarly, Yin et al. confirmed the presence of TRPV4 proteins in both murine and human neutrophils. Depletion of TRPV4 attenuates platelet-activating factor-induced elevation of intracellular calcium levels, preventing neutrophils from recognizing pro-inflammatory stimuli such as ROS formation, adhesion, and chemotaxis [82].

In macrophages, TRPM2 is essential for the activation of CD36 by its ligands, including oxidized low-density lipoprotein and thrombospondin-1. Suppression of TRPM2 in mice has the potential to prevent atherosclerosis by disrupting the TRPM2–CD36 inflammatory axis in macrophages [27]. IFN-γ stimulation can induce TRPM2 activation in microglia, leading to excessive production of ROS, which further enhances the generation of apoptotic proteases, such as caspase-3 and -9, as well as pro-inflammatory factors, including TNF-α, IL-1β, and IL-6 [41]. Calcium signaling induced by inflammation facilitates microglia activation and the subsequent release of neurotoxic agents (pro-inflammatory cytokines and NO), ultimately resulting in neuronal degeneration. Upon exposure to LPS/IFN-γ, TRPM2 transitions into an open state and orchestrates downstream p38 MAPK and JNK signaling pathways, thereby promoting a burst of NO production [83]. TRPM2 also plays a crucial role in mediating neutrophil migration and promoting vascular injury. Under conditions of oxidative stress, hydrogen peroxide (H_2_O_2_) triggers the activation of adipocyte differentiation-related protein (ADRP) within the nucleus and mitochondria, subsequently stimulating TRPM2. Furthermore, polymorphocytic neutrophils (PMNs) and endothelial cells (ECs) coordinate and interact to facilitate PMN migration. The generation of ROS by PMN is responsible for essential TRPM2 activation in EC. By permeating calcium ions into ECs, TRPM2 disrupts cadherin junctions to mediate PMN migration across the vascular endothelial barrier towards the infection site, combating inflammation [51]. Additionally, recent research highlights the critical role of TRPM2 in regulating DC chemotaxis through Ca^2+^ release [54].

Activation of the NLRP3 inflammasome in microglia amplifies neuroinflammation in a rat model of cardiac arrest and cardiopulmonary resuscitation. The sequential activation of SUR1-TRPM4 and the NLRP3 inflammasome is known to effectively respond to damage, stress, and neuroinflammation in microglia. Therefore, inhibiting SUR1-TRPM4 may provide an anti-inflammatory and neuroprotective effect for preventing brain edema [42]. TRPM5 has been exclusively detected in murine tracheal brush cells, where its activation induces neurogenic inflammation followed by neutrophil recruitment. Notably, knockout of Trpm5 results in a significant reduction in pro-inflammatory factors, such as IL-1α, IL-6, KC (a CXC chemokine), monocyte chemoattractant protein (MCP)-1, granulocyte colony-stimulating factor (G-CSF), and eotaxin (a CC chemokine), in infected Trpm5^−/−^ mice [84].

Calcium signaling serves as the principle mechanism by which TRPM7 contributes to pro-inflammatory processes. Additionally, TRPM7-induced calcium influx co-regulates calmodulin-dependent protein kinase II (CAMKII) and tumor necrosis factor receptor-associated factor 6 (TRAF6), leading to the activation of TGF-β activated kinase 1 and NF-κB, thereby enhancing adipose inflammation [85]. Intracellular Ca^2+^ is involved in mast cell degranulation and the promotion of pro-inflammatory responses. Notably, a study demonstrated that intracellular Mg^2+^ also plays a critical role in mast cell survival through knockdown of Trpm7 [46]. Deletion of the TRPM7 gene results in an increase in IL-2 and IL-2Rα within T cells, highlighting its involvement in their lineage development. The enrichment of IL-2 signaling evokes STAT5 activation, a key factor for Foxp3 transcriptional regulation. Consequently, increased expression of Foxp3 promotes thymic Treg cell development while restraining local expansion of conventional T cells into damage-causing Teff cells [63]. Notably, cyclooxygenase-2 (COX-2) is up-regulated in inflamed tissues and plays a crucial role in both the initiation and resolution of inflammation. Inhibition of TRPM7 kinase activity effectively suppresses AKT phosphorylation and subsequently reduces COX-2 expression in peripheral blood mononuclear cells. These findings highlight the potent impact of TRPM7 kinase on the AKT-driven signaling pathway, suggesting that therapeutic intervention targeting TRPM7 may facilitate recovery from COX-2-mediated inflammation [86]. Furthermore, it has been demonstrated that TRPM7 kinase modulates neutrophil recruitment through activation of AKT/mTOR pathways [52].

TRPML1 is an essential factor for the rapid motility, chemotaxis, and migration of DCs in vivo [57]. Agonistic stimulation of TRPML ameliorates specific degranulation or IFN-γ responses in resting primary NK cells [69]. Activators of TRPML2 selectively induce CCL2 secretion in macrophages expressing TRPML2, subsequently recruiting more macrophages to modulate the inflammatory response [35]. Notably, Trpml2 knockout mice exhibit a significant reduction in macrophage recruitment upon LPS stimulation. Furthermore, Trpml2^−/−^ animals demonstrate a substantial decrease in neutrophil migration to the intraperitoneal space [87].

### 3.2. Anti-Inflammatory Effects of TRPs

**Figure 2:**
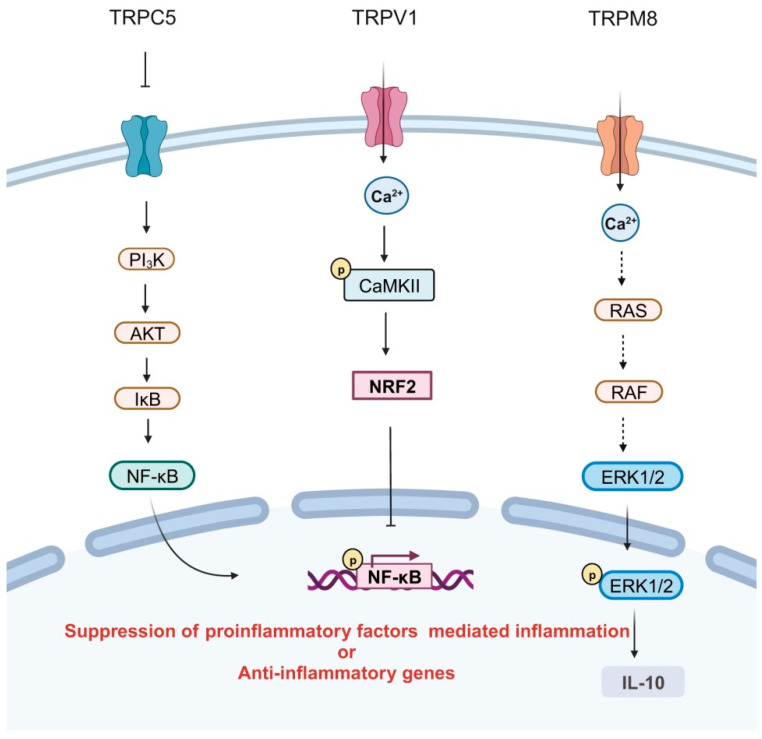
TRPs modulate anti-inflammation in immune cells. Suppression of TRPC5 activates the PI3K/AKT/IκB pathway, leading to subsequent phosphorylation of NF-κB and thereby promoting the release of pro-inflammatory factors. Activation of TRPV1 facilitates calmodulin-dependent protein kinase II (CaMKII) phosphorylation, which promotes NRF2 nuclear translocation and inhibits the release of pro-inflammatory factors. Activation of TRPM8 induces ERK1/2 phosphorylation, resulting in the release of the anti-inflammatory factor IL-10.

An increasing number of studies have unveiled that TRP ion channels, when expressed in certain immune cells, demonstrate potent anti-inflammatory properties under specific circumstances (Figure 2). BDNF induces a sustained elevation of intracellular Ca^2+^ by maintaining the activation of TRPC3 channels in rodent microglia, thereby enabling microglial cells to effectively respond to inflammatory stimuli through down-regulation of TNF-α [39]. Tao et al. discovered that suppression of TRPC5 enhances the production of TNF-α, IL-6, and other inflammatory cytokines in the peripheral blood of mice. Furthermore, activation of TRPC5 may confer an anti-inflammatory effect by inhibiting macrophage polarization towards the M1 phenotype [21].

TRPA1 negatively regulates the transition of macrophages to an M1 phenotype in the mouse kidney, leading to an enrichment of pro-inflammatory factors such as IL-1β and TNF-α [88]. Bertin et al. have demonstrated that CD4^+^ T cells isolated from IL10^−/−^ Trpa1^−/−^ mice exhibit a Th1-mediated inflammatory profile characterized by the production of Th1-type cytokines IFN-γ and IL-2 [89]. Furthermore, TRPA1 expression increases during T cell activation, where specifically activated TRPA1 restrains the secretion of pro-inflammatory cytokines, including TNF-α, IFN-γ, and IL-2, through elevated calcium influx mediated by TRPA1 in T cells [90].

Capsaicin serves as a suppressor for M1 macrophage polarization via TRPV1 activation. The inflammatory mediators IL-1β, IL-6, IL-8, IL-18, TNF-α, and iNOS are diminished by TRPV1 via the Ca^2+^/CaMKII/nuclear factor erythroid-2-related factor 2 (NRF2) signaling pathway [22]. Selective deletion of TRPV1 in dermal macrophages results in excessive accumulation of TNF-α, IL-1β, and IL-6, leading to an exaggerated inflammatory response [91]. Recently validated by Ueno et al., Trpv1^−/−^ mice exhibit reduced macrophage presence during inflammation after injury. In Trpv1 KO mice, there is a prolonged neutrophilic inflammatory response and excessive formation of neutrophil extracellular traps (NETs), thereby impeding the wound healing process [92].

The TRPV4 ion channel acts as a thermosensor, detecting changes in temperature. Activation of TRPV4 elevates mitochondrial calcium levels, which play a crucial role in regulating the morphology of immune cells and the formation of synapses in murine T cells. Consequently, the presence of TRPV4 within mitochondria facilitates both T cell activation and migration. The signaling pathway mediated by TRPV4 provides a protective mechanism that prevents excessive stimulation of T cells and subsequent cellular damage [66].

TRPM2 activation triggers plasma membrane depolarization in phagocytes, leading to a reduction in NADPH oxidation-mediated ROS production [93]. Beceiro et al. demonstrated that Trpm2 deficiency in macrophages results in calcium overload, which enhances MAPK signaling, modulates NADPH oxidase activities, and promotes the release of inflammatory factors, thereby exacerbating gastric mucosa inflammation [29].

The mast cell plays a crucial role in the development of allergies. Upon allergic stimulation, Trpm4^−/−^ mast cells exhibit heightened calcium signaling transduction, enhanced degranulation, and increased production of histamine leukotrienes and tumor necrosis factor and trigger a pronounced IgE-mediated acute passive skin sensitization inflammatory response [45]. Emerging evidence suggests that suppression of TRPM4 leads to an elevation in calcium influx, which is associated with the recruitment of pro-inflammatory neutrophils, M1 monocytes, and macrophages as well as the transcription of intricate pro-inflammatory genes [94]. The distribution of TRPM4 has been found to be higher in Th2 cells compared to Th1 cells. Inhibition of TRPM4 in Th2 cells leads to an increase in Ca^2+^ influx and release of IL-2 while also decreasing cell motility. Conversely, inhibition of TRPM4 in Th1 cells results in a high-mobility group with a decline in Ca^2+^ influx as well as reduced production of IL-2 and IFN-γ [95]. LPS-stimulated B cells from Trpm5-deficient mice demonstrate elevated intracellular calcium levels and secrete the inflammatory cytokines IL-6 and CXCL-10 [68].

TRPM7 current exhibits a significantly greater magnitude in anti-inflammatory M2-type macrophages compared to pro-inflammatory M1-type macrophages, while the mRNA levels of TRPM7 remain unchanged during cell polarization. Pharmacological inhibition of TRPM7 by its specific inhibitors of NS8593 and FTY720 impedes the polarization process towards an M2 phenotype in macrophages [96]. Activation of TRPM8 elicits an anti-inflammatory cytokine profile in macrophages, whereas Trpm8-deficient macrophages exacerbate inflammation [34].

The modulation of TRPV1 and TRPA1 by CBC has been validated, highlighting their potential as therapeutic targets [36]. Additionally, the anti-inflammatory effects of CBC in activated macrophages further support this notion [37]. Moreover, it has been suggested that TRPA1 may play a role in mitigating the risk of ischemia-reperfusion-induced acute kidney injury via its orchestration of the macrophage-mediated inflammatory pathway [88].

### 3.3. The Paradoxical Roles of TRPs in Inflammation

TRP ion channels can exhibit divergent roles in inflammation, either promoting or inhibiting the inflammatory response depending on their distribution within distinct immune cell populations. Specifically, resident TRPC3 in macrophages is associated with a pro-inflammatory effect, while its presence in microglia and T lymphocytes contributes to an anti-inflammatory response [20,39,65]. TRPM2 is widely expressed in macrophages, microglial cells, neutrophils, and DCs. It functions as a pro-inflammatory factor in microglia, neutrophils, and DCs [41,51,54]. In macrophages, it confers an anti-inflammatory effect but exhibits a pro-inflammatory response concomitant with CD36 activation [27,29]. TRPM4 acts as a pro-inflammatory factor in microglia but as an anti-inflammatory factor in macrophages, mast cells, and T lymphocytes [42,45,94,95]. TRPM7 has been identified as being widely expressed in macrophages, mast cells, neutrophils, and T lymphocytes. It demonstrates an anti-inflammatory phenotype in macrophages while exerting pro-inflammatory roles in mast cells and T cells [46,52,63,96]. TRPA1 is expressed in macrophages, microglia, and T lymphocytes. It confers an anti-inflammatory effect in T cells and macrophages but a pro-inflammatory effect in microglia [37,78,90]. TRPV1 exerts anti-inflammatory effects in macrophages and neutrophils but pro-inflammatory effects in microglia [22,40,91,92]. Further, TRPV4 plays a pro-inflammatory role in neutrophils, but in T lymphocytes, it exhibits an anti-inflammatory effect [50,66].

## 4. Conclusions and Outlook

TRP ion channels distributed in various immune cells extend their function as nociceptors in both physiological and pathological conditions. Growing evidence supports the involvement of TRP ion channels in certain diseases through inflammatory responses, including sepsis, colitis, pain, itch, allergy, asthma, atherosclerosis, and Alzheimer’s disease [28,37,62,77,97,98]. Moreover, TRP ion channels have also been implicated in obesity and diabetes as well as certain types of cancer via inflammation [5,64,85,99,100]. Recent studies have highlighted the participation of TRP ion channels in the infection process of severe acute respiratory syndrome coronavirus-2 (SARS-CoV-2), suggesting their potential as therapeutic targets for SARS-CoV-2 and COVID-19 (coronavirus disease 2019) [24,101,102,103]. Therefore, when targeting TRPs for intervention against inflammation in these diseases, it is crucial to consider the pro-inflammatory or anti-inflammatory functions exhibited by TRPs within immune cells and choose whether to activate or inhibit TRP ion channels accordingly to suppress inflammation flare-ups.

Significant progress has been made on the involvement of TRPs in pro- and anti-inflammatory responses, but a more precise mechanism remains elusive. TRP ion channels are involved in pro-inflammatory and anti-inflammatory processes by mediating calcium signaling [19,22,26,32,54,66,76]. The dynamic distribution of TRP ion channels upon stimuli influences intracellular calcium signaling, making the regulatory process more complicated and unpredictable. Cytosolic calcium signaling not only regulates cell proliferation and death but also participates in different transcriptional processes [22,66,76,95]. Thus, exploration of the pro-inflammatory or anti-inflammatory mechanisms mediated by calcium signals in the dynamic changes is worthy of in-depth consideration.

As cell membrane receptors, TRP ion channels can be regulated by a diverse range of physical stimuli and chemical agents as well as endogenous ligands. Many of these factors coincide with inflammatory elements [12,20,78,83,93]. It has been suggested that there are an abundance of intervention options available at this juncture. TRP ion channels consist of several conserved structural elements, including the ankyrin repeat domain, TRP box, and coiled-coil domain [10,11]. These specific domains facilitate interactions between TRPs and other proteins that trigger downstream cascades involved in inflammation and immunity. Therefore, deciphering the regulation of TRP ion channels in inflammation will yield new insights for interventions targeting inflammation and even immunotherapy for related diseases.

## Figures and Tables

**Table 1:** Distribution of TRPs in immune system cells.

Immune Cells	TRP Channels	Reference
Macrophages	TRPA1 TRPML2 TRPC1, TRPC3, TRPC5 TRPV1, TRPV2, TRPV4 TRPM2, TRPM4, TRPM7, TRPM8	[37] [35] [19,20,21] [22,23,24,25,26] [27,28,29,30,31,32,33,34]
Microglia	TRPC3 TRPV1 TRPM2, TRPM4	[39] [40] [41,42]
Neutrophils	TRPV4 TRPC1, TRPC6 TRPM2, TRPM7	[50] [48,49] [51,52]
Dendritic cells	TRPV1 TRPM2 TRPML1	[55,56] [54] [57]
Mast cells	TRPM4, TRPM7	[45,46]
B lymphocytes	TRPM5	[68]
T lymphocytes	TRPA1,TRPV1 TRPV4 TRPM7 TRPC3, TRPC6	[61] [66] [31,63] [62,65]
Natural killer cells	TRPML1	[69]

## Data Availability

Data is contained within the article.

## Abbreviations

TRP: transient receptor potential; WT: wide-type; DCs: dendritic cells; NK: natural killer; LPS: lipopolysaccharide; CHIKV: Chikungunya virus; AMs: alveolar macrophages; CBC: cannabichromene; BDNF: brain-derived neurotrophic factor; IFN-γ: interferon-γ; SUR1: sulfonylurea receptor 1; DAMPs: damage-associated molecular patterns; ROS: reactive oxygen species; Th cells: T helper cells; IL: interleukin; TNF: tumor necrosis factor; NF-κB: nuclear factor-κB; MMP9: matrix metalloprotease 9; PMN: polymorphocytic neutrophils; EC: endothelial cells; CAMKII: calmodulin-dependent protein kinase II; TRAF6: tumor necrosis factor receptor-associated factor 6; COX-2: cyclooxygenase-2; NRF2: nuclear factor erythroid-2-related factor 2; ATP: adenosine triphosphate; CCR7: CC chemokine receptor 7; CCL19: CC chemokine ligand 19; CCL21: CC chemokine ligand 21; Treg: regulatory T; Tfh: follicular helper T; NETs: neutrophil extracellular traps; MHC: major histocompatibility complex; TLR4: toll-like receptor 4; TGF-β: transforming growth factor-β; STAT: signal transducer and activator of transcription; ER: endoplasmic reticulum; PA: phosphatidic acid; DAG: diacylglycerol; CXCR2: CXC motif chemokine receptor 2; AKT: protein kinase B; MAPK: mitogen-activated protein kinase; JNK: c-Jun N-terminal kinase; NLRP3: NACHT, LRR, and PYD domains-containing protein 3; cAMP: cyclic adenosine monophosphate; ADRP: adipocyte differentiation-related protein; MCP-1: monocyte chemoattractant protein-1; G-CSF: granulocyte colony-stimulating factor; Foxp3: forkhead box protein P3; mTOR: mammalian target of rapamycin; PKC: protein kinase C; PP2A: protein phosphatase 2A; NAPDH: nicotinamide adenine dinucleotide phosphate; iNOS: inducible nitric oxide synthase; PI3K: phosphatidylinositol 3 kinase; ERK1/2: extracellular signal-regulated kinase 1 and 2; CD36: cluster of differentiation 36; SARS-CoV-2: severe acute respiratory syndrome coronavirus-2; COVID-19: coronavirus disease 2019.

## References

[1] Leigh T., Scalia R.G., Autieri M.V. (2020). Resolution of inflammation in immune and nonimmune cells by interleukin-19. Am. J. Physiol. Cell Physiol..

[2] Caballero-Sanchez N., Alonso-Alonso S., Nagy L. (2022). Regenerative inflammation: When immune cells help to re-build tissues. FEBS J..

[3] Asano H., Hasegawa-Ishii S., Arae K., Obara A., Laumet G., Dantzer R., Shimada A. (2022). Infiltration of peripheral immune cells into the olfactory bulb in a mouse model of acute nasal inflammation. J. Neuroimmunol..

[4] Pillon N.J., Smith J.A.B., Alm P.S., Chibalin A.V., Alhusen J., Arner E., Carninci P., Fritz T., Otten J., Olsson T. (2022). Distinctive exercise-induced inflammatory response and exerkine induction in skeletal muscle of people with type 2 diabetes. Sci. Adv..

[5] Chang Z., Li R., Zhang J., An L., Zhou G., Lei M., Deng J., Yang R., Song Z., Zhong W. (2022). Distinct immune and inflammatory response patterns contribute to the identification of poor prognosis and advanced clinical characters in bladder cancer patients. Front. Immunol..

[6] Li C., Gao Q., Jiang H., Liu C., Du Y., Li L. (2022). Changes of macrophage and CD4^+^ T cell in inflammatory response in type 1 diabetic mice. Sci. Rep..

[7] Shaker T., Chattopadhyaya B., Amilhon B., Cristo G.D., Weil A.G. (2021). Transduction of inflammation from peripheral immune cells to the hippocampus induces neuronal hyperexcitability mediated by Caspase-1 activation. Neurobiol. Dis..

[8] Rustenhoven J., Kipnis J. (2019). Smelling Danger: Olfactory Stem Cells Control Immune Defense during Chronic Inflammation. Cell Stem Cell.

[9] Chen X., Jaiswal A., Costliow Z., Herbst P., Creasey E.A., Oshiro-Rapley N., Daly M.J., Carey K.L., Graham D.B., Xavier R.J. (2022). pH sensing controls tissue inflammation by modulating cellular metabolism and endo-lysosomal function of immune cells. Nat. Immunol..

[10] Cheng W., Zheng J. (2021). Distribution and Assembly of TRP Ion Channels. Adv. Exp. Med. Biol..

[11] Nilius B., Flockerzi V. (2014). Mammalian transient receptor potential (TRP) cation channels. Preface. Handb. Exp. Pharmacol..

[12] Zhao J., Lin King J.V., Paulsen C.E., Cheng Y., Julius D. (2020). Irritant-evoked activation and calcium modulation of the TRPA1 receptor. Nature.

[13] Earley S., Santana L.F., Lederer W.J. (2022). The physiological sensor channels TRP and piezo: Nobel Prize in Physiology or Medicine 2021. Physiol. Rev..

[14] Diaz-Franulic I., Raddatz N., Castillo K., Gonzalez-Nilo F.D., Latorre R. (2020). A folding reaction at the C-terminal domain drives temperature sensing in TRPM8 channels. Proc. Natl. Acad. Sci. USA.

[15] Song K., Wang H., Kamm G.B., Pohle J., Reis F.C., Heppenstall P., Wende H., Siemens J. (2016). The TRPM2 channel is a hypothalamic heat sensor that limits fever and can drive hypothermia. Science.

[16] Yin Y., Wu M., Zubcevic L., Borschel W.F., Lander G.C., Lee S.Y. (2018). Structure of the cold- and menthol-sensing ion channel TRPM8. Science.

[17] Nam J.H., Kim W.K. (2020). The Role of TRP Channels in Allergic Inflammation and its Clinical Relevance. Curr. Med. Chem..

[18] Khalil M., Alliger K., Weidinger C., Yerinde C., Wirtz S., Becker C., Engel M.A. (2018). Functional Role of Transient Receptor Potential Channels in Immune Cells and Epithelia. Front. Immunol..

[19] Nascimento Da Conceicao V., Sun Y., Ramachandran K., Chauhan A., Raveendran A., Venkatesan M., DeKumar B., Maity S., Vishnu N., Kotsakis G.A. (2021). Resolving macrophage polarization through distinct Ca^2+^ entry channel that maintains intracellular signaling and mitochondrial bioenergetics. iScience.

[20] Casas J., Meana C., López-López J.R., Balsinde J., Balboa M.A. (2021). Lipin-1-derived diacylglycerol activates intracellular TRPC3 which is critical for inflammatory signaling. Cell. Mol. Life Sci..

[21] Tao L., Guo G., Qi Y., Xiong Y., Ma X., Wu N., Dong C., Yang C. (2020). Inhibition of Canonical Transient Receptor Potential 5 Channels Polarizes Macrophages to an M1 Phenotype. Pharmacology.

[22] Lv Z., Xu X., Sun Z., Yang Y.X., Guo H., Li J., Sun K., Wu R., Xu J., Jiang Q. (2021). TRPV1 alleviates osteoarthritis by inhibiting M1 macrophage polarization via Ca^2+^/CaMKII/Nrf2 signaling pathway. Cell Death Dis..

[23] Sanjai Kumar P., Nayak T.K., Mahish C., Sahoo S.S., Radhakrishnan A., De S., Datey A., Sahu R.P., Goswami C., Chattopadhyay S. (2021). Inhibition of transient receptor potential vanilloid 1 (TRPV1) channel regulates chikungunya virus infection in macrophages. Arch. Virol..

[24] Xu J., Yang Y., Hou Z., Jia H., Wang Y. (2021). TRPV2-spike protein interaction mediates the entry of SARS-CoV-2 into macrophages in febrile conditions. Theranostics.

[25] Raudszus R., Paulig A., Urban N., Deckers A., Grassle S., Vanderheiden S., Jung N., Brase S., Schaefer M., Hill K. (2023). Pharmacological inhibition of TRPV2 attenuates phagocytosis and lipopolysaccharide-induced migration of primary macrophages. Br. J. Pharmacol..

[26] Rayees S., Joshi J.C., Tauseef M., Anwar M., Baweja S., Rochford I., Joshi B., Hollenberg M.D., Reddy S.P., Mehta D. (2019). PAR2-Mediated cAMP Generation Suppresses TRPV4-Dependent Ca^2+^ Signaling in Alveolar Macrophages to Resolve TLR4-Induced Inflammation. Cell Rep..

[27] Zong P., Feng J., Yue Z., Yu A.S., Vacher J., Jellison E.R., Miller B., Mori Y., Yue L. (2022). TRPM2 deficiency in mice protects against atherosclerosis by inhibiting TRPM2-CD36 inflammatory axis in macrophages. Nat. Cardiovasc. Res..

[28] Zhang Y., Ying F., Tian X., Lei Z., Li X., Lo C.Y., Li J., Jiang L., Yao X. (2022). TRPM2 Promotes Atherosclerotic Progression in a Mouse Model of Atherosclerosis. Cells.

[29] Beceiro S., Radin J.N., Chatuvedi R., Piazuelo M.B., Horvarth D.J., Cortado H., Gu Y., Dixon B., Gu C., Lange I. (2017). TRPM2 ion channels regulate macrophage polarization and gastric inflammation during Helicobacter pylori infection. Mucosal Immunol..

[30] Serafini N., Dahdah A., Barbet G., Demion M., Attout T., Gautier G., Arcos-Fajardo M., Souchet H., Jouvin M.H., Vrtovsnik F. (2012). The TRPM4 channel controls monocyte and macrophage, but not neutrophil, function for survival in sepsis. J. Immunol..

[31] Romagnani A., Vettore V., Rezzonico-Jost T., Hampe S., Rottoli E., Nadolni W., Perotti M., Meier M.A., Hermanns C., Geiger S. (2017). TRPM7 kinase activity is essential for T cell colonization and alloreactivity in the gut. Nat. Commun..

[32] Schappe M.S., Stremska M.E., Busey G.W., Downs T.K., Seegren P.V., Mendu S.K., Flegal Z., Doyle C.A., Stipes E.J., Desai B.N. (2022). Efferocytosis requires periphagosomal Ca^2+^-signaling and TRPM7-mediated electrical activity. Nat. Commun..

[33] Qiao W., Wong K.H.M., Shen J., Wang W., Wu J., Li J., Lin Z., Chen Z., Matinlinna J.P., Zheng Y. (2021). TRPM7 kinase-mediated immunomodulation in macrophage plays a central role in magnesium ion-induced bone regeneration. Nat. Commun..

[34] Khalil M., Babes A., Lakra R., Försch S., Reeh P.W., Wirtz S., Becker C., Neurath M.F., Engel M.A. (2016). Transient receptor potential melastatin 8 ion channel in macrophages modulates colitis through a balance-shift in TNF-alpha and interleukin-10 production. Mucosal Immunol..

[35] Plesch E., Chen C.C., Butz E., Scotto Rosato A., Krogsaeter E.K., Yinan H., Bartel K., Keller M., Robaa D., Teupser D. (2018). Selective agonist of TRPML2 reveals direct role in chemokine release from innate immune cells. eLife.

[36] Maione S., Piscitelli F., Gatta L., Vita D., De Petrocellis L., Palazzo E., de Novellis V., Di Marzo V. (2011). Non-psychoactive cannabinoids modulate the descending pathway of antinociception in anaesthetized rats through several mechanisms of action. Br. J. Pharmacol..

[37] Romano B., Borrelli F., Fasolino I., Capasso R., Piscitelli F., Cascio M., Pertwee R., Coppola D., Vassallo L., Orlando P. (2013). The cannabinoid TRPA1 agonist cannabichromene inhibits nitric oxide production in macrophages and ameliorates murine colitis. Br. J. Pharmacol..

[38] Borst K., Dumas A.A., Prinz M. (2021). Microglia: Immune and non-immune functions. Immunity.

[39] Mizoguchi Y., Monji A. (2017). TRPC Channels and Brain Inflammation. Adv. Exp. Med. Biol..

[40] Zhang Y., Hou B., Liang P., Lu X., Wu Y., Zhang X., Fan Y., Liu Y., Chen T., Liu W. (2021). TRPV1 channel mediates NLRP3 inflammasome-dependent neuroinflammation in microglia. Cell Death Dis..

[41] Akyuva Y., Nazıroğlu M., Yıldızhan K. (2021). Selenium prevents interferon-gamma induced activation of TRPM2 channel and inhibits inflammation, mitochondrial oxidative stress, and apoptosis in microglia. Metab. Brain Dis..

[42] He Y., Chang Y., Peng Y., Zhu J., Liu K., Chen J., Wu Y., Ji Z., Lin Z., Wang S. (2022). Glibenclamide Directly Prevents Neuroinflammation by Targeting SUR1-TRPM4-Mediated NLRP3 Inflammasome Activation In Microglia. Mol. Neurobiol..

[43] Conti P., Lauritano D., Caraffa A., Gallenga C.E., Kritas S.K., Ronconi G., Pandolfi F. (2019). New insight into systemic mastocytosis mediated by cytokines IL-1beta and IL-33: Potential inhibitory effect of IL-37. Eur. J. Pharmacol..

[44] Wu C., Boey D., Bril O., Grootens J., Vijayabaskar M.S., Sorini C., Ekoff M., Wilson N.K., Ungerstedt J.S., Nilsson G. (2022). Single-cell transcriptomics reveals the identity and regulators of human mast cell progenitors. Blood Adv..

[45] Vennekens R., Olausson J., Meissner M., Bloch W., Mathar I., Philipp S.E., Schmitz F., Weissgerber P., Nilius B., Flockerzi V. (2007). Increased IgE-dependent mast cell activation and anaphylactic responses in mice lacking the calcium-activated nonselective cation channel TRPM4. Nat. Immunol..

[46] Wykes R.C., Lee M., Duffy S.M., Yang W., Seward E.P., Bradding P. (2007). Functional transient receptor potential melastatin 7 channels are critical for human mast cell survival. J. Immunol..

[47] Castanheira F.V.S., Kubes P. (2019). Neutrophils and NETs in modulating acute and chronic inflammation. Blood.

[48] Lindemann O., Strodthoff C., Horstmann M., Nielsen N., Jung F., Schimmelpfennig S., Heitzmann M., Schwab A. (2015). TRPC1 regulates fMLP-stimulated migration and chemotaxis of neutrophil granulocytes. Biochim. Biophys. Acta.

[49] Lindemann O., Umlauf D., Frank S., Schimmelpfennig S., Bertrand J., Pap T., Hanley P.J., Fabian A., Dietrich A., Schwab A. (2013). TRPC6 regulates CXCR2-mediated chemotaxis of murine neutrophils. J. Immunol..

[50] Wu Y., Lu K., Lu Y., Liao J., Zhang S., Yang S., Zhao N., Dong Q., Chen L., Wu Q. (2023). Transient receptor potential vanilloid 4 (TRPV4) in neutrophils enhances myocardial ischemia/reperfusion injury. J. Leukoc. Biol..

[51] Mittal M., Nepal S., Tsukasaki Y., Hecquet C.M., Soni D., Rehman J., Tiruppathi C., Malik A.B. (2017). Neutrophil Activation of Endothelial Cell-Expressed TRPM2 Mediates Transendothelial Neutrophil Migration and Vascular Injury. Circ. Res..

[52] Nadolni W., Immler R., Hoelting K., Fraticelli M., Ripphahn M., Rothmiller S., Matsushita M., Boekhoff I., Gudermann T., Sperandio M. (2020). TRPM7 Kinase Is Essential for Neutrophil Recruitment and Function via Regulation of Akt/mTOR Signaling. Front. Immunol..

[53] Liu Z., Roche P.A. (2015). Macropinocytosis in phagocytes: Regulation of MHC class-II-restricted antigen presentation in dendritic cells. Front. Physiol..

[54] Sumoza-Toledo A., Lange I., Cortado H., Bhagat H., Mori Y., Fleig A., Penner R., Partida-Sánchez S. (2011). Dendritic cell maturation and chemotaxis is regulated by TRPM2-mediated lysosomal Ca^2+^ release. FASEB J..

[55] Assas M.B., Wakid M.H., Zakai H.A., Miyan J.A., Pennock J.L. (2016). Transient receptor potential vanilloid 1 expression and function in splenic dendritic cells: A potential role in immune homeostasis. Immunology.

[56] Yao E., Zhang G., Huang J., Yang X., Peng L., Huang X., Luo X., Ren J., Huang R., Yang L. (2019). Immunomodulatory effect of oleoylethanolamide in dendritic cells via TRPV1/AMPK activation. J. Cell. Physiol..

[57] Bretou M., Sáez P.J., Sanséau D., Maurin M., Lankar D., Chabaud M., Spampanato C., Malbec O., Barbier L., Muallem S. (2017). Lysosome signaling controls the migration of dendritic cells. Sci. Immunol..

[58] MartIn-Fontecha A., Sebastiani S., Höpken U.E., Uguccioni M., Lipp M., Lanzavecchia A., Sallusto F. (2003). Regulation of dendritic cell migration to the draining lymph node: Impact on T lymphocyte traffic and priming. J. Exp. Med..

[59] Weber M., Hauschild R., Schwarz J., Moussion C., de Vries I., Legler D.F., Luther S.A., Bollenbach T., Sixt M. (2013). Interstitial dendritic cell guidance by haptotactic chemokine gradients. Science.

[60] Saravia J., Chapman N.M., Chi H. (2019). Helper T cell differentiation. Cell. Mol. Immunol..

[61] Cevikbas F., Wang X., Akiyama T., Kempkes C., Savinko T., Antal A., Kukova G., Buhl T., Ikoma A., Buddenkotte J. (2014). A sensory neuron-expressed IL-31 receptor mediates T helper cell-dependent itch: Involvement of TRPV1 and TRPA1. J. Allergy Clin. Immunol..

[62] Sel S., Rost B.R., Yildirim A.O., Sel B., Kalwa H., Fehrenbach H., Renz H., Gudermann T., Dietrich A. (2008). Loss of classical transient receptor potential 6 channel reduces allergic airway response. Clin. Exp. Allergy.

[63] Mendu S.K., Stremska M.E., Schappe M.S., Moser E.K., Krupa J.K., Rogers J.S., Stipes E.J., Parker C.A., Braciale T.J., Perry J.S.A. (2020). Targeting the ion channel TRPM7 promotes the thymic development of regulatory T cells by promoting IL-2 signaling. Sci. Signal..

[64] Reina-Campos M., Scharping N.E., Goldrath A.W. (2021). CD8^+^ T cell metabolism in infection and cancer. Nat. Rev. Immunol..

[65] Jing C., Dongming Z., Hong C., Quan N., Sishi L., Caixia L. (2018). TRPC3 Overexpression Promotes the Progression of Inflammation-Induced Preterm Labor and Inhibits T Cell Activation. Cell. Physiol. Biochem..

[66] Acharya T.K., Kumar S., Rokade T.P., Chang Y.T., Goswami C. (2023). TRPV4 regulates mitochondrial Ca^2+^-status and physiology in primary murine T cells based on their immunological state. Life Sci..

[67] Seifert M., Küppers R. (2016). Human memory B cells. Leukemia.

[68] Sakaguchi T., Okumura R., Ono C., Okuzaki D., Kawai T., Okochi Y., Tanimura N., Murakami M., Kayama H., Umemoto E. (2020). TRPM5 Negatively Regulates Calcium-Dependent Responses in Lipopolysaccharide-Stimulated B Lymphocytes. Cell Rep..

[69] Goodridge J.P., Jacobs B., Saetersmoen M.L., Clement D., Hammer Q., Clancy T., Skarpen E., Brech A., Landskron J., Grimm C. (2019). Remodeling of secretory lysosomes during education tunes functional potential in NK cells. Nat. Commun..

[70] Jarczak D., Nierhaus A. (2022). Cytokine Storm-Definition, Causes, and Implications. Int. J. Mol. Sci..

[71] Roe K. (2021). An inflammation classification system using cytokine parameters. Scand. J. Immunol..

[72] Medzhitov R. (2021). The spectrum of inflammatory responses. Science.

[73] Bhat A.A., Nisar S., Singh M., Ashraf B., Masoodi T., Prasad C.P., Sharma A., Maacha S., Karedath T., Hashem S. (2022). Cytokine- and chemokine-induced inflammatory colorectal tumor microenvironment: Emerging avenue for targeted therapy. Cancer Commun..

[74] Fajgenbaum D.C., June C.H. (2020). Cytokine Storm. N. Engl. J. Med..

[75] Park J.H., Lee D.H., Park M.S., Jung Y.S., Hong J.T. (2017). C-C chemokine receptor type 5 deficiency exacerbates alcoholic fatty liver disease through pro-inflammatory cytokines and chemokines-induced hepatic inflammation. J. Gastroenterol. Hepatol..

[76] Chen Q., Zhou Y., Zhou L., Fu Z., Yang C., Zhao L., Li S., Chen Y., Wu Y., Ling Z. (2020). TRPC6-dependent Ca^2+^ signaling mediates airway inflammation in response to oxidative stress via ERK pathway. Cell Death Dis..

[77] Lee K.I., Lee H.T., Lin H.C., Tsay H.J., Tsai F.C., Shyue S.K., Lee T.S. (2016). Role of transient receptor potential ankyrin 1 channels in Alzheimer’s disease. J. Neuroinflamm..

[78] Yap J.M.G., Ueda T., Takeda N., Fukumitsu K., Fukuda S., Uemura T., Tajiri T., Ohkubo H., Maeno K., Ito Y. (2020). An inflammatory stimulus sensitizes TRPA1 channel to increase cytokine release in human lung fibroblasts. Cytokine.

[79] Ninomiya Y., Tanuma S.I., Tsukimoto M. (2017). Differences in the effects of four TRPV1 channel antagonists on lipopolysaccharide-induced cytokine production and COX-2 expression in murine macrophages. Biochem Biophys Res Commun.

[80] Yamashiro K., Sasano T., Tojo K., Namekata I., Kurokawa J., Sawada N., Suganami T., Kamei Y., Tanaka H., Tajima N. (2010). Role of transient receptor potential vanilloid 2 in LPS-induced cytokine production in macrophages. Biochem. Biophys. Res. Commun..

[81] Dutta B., Goswami R., Rahaman S.O. (2020). TRPV4 Plays a Role in Matrix Stiffness-Induced Macrophage Polarization. Front. Immunol..

[82] Yin J., Michalick L., Tang C., Tabuchi A., Goldenberg N., Dan Q., Awwad K., Wang L., Erfinanda L., Nouailles G. (2016). Role of Transient Receptor Potential Vanilloid 4 in Neutrophil Activation and Acute Lung Injury. Am. J. Respir. Cell Mol. Biol..

[83] Miyake T., Shirakawa H., Kusano A., Sakimoto S., Konno M., Nakagawa T., Mori Y., Kaneko S. (2014). TRPM2 contributes to LPS/IFNγ-induced production of nitric oxide via the p38/JNK pathway in microglia. Biochem. Biophys. Res. Commun..

[84] Hollenhorst M.I., Nandigama R., Evers S.B., Gamayun I., Abdel Wadood N., Salah A., Pieper M., Wyatt A., Stukalov A., Gebhardt A. (2022). Bitter taste signaling in tracheal epithelial brush cells elicits innate immune responses to bacterial infection. J. Clin. Investig..

[85] Zhong W., Ma M., Xie J., Huang C., Li X., Gao M. (2023). Adipose-specific deletion of the cation channel TRPM7 inhibits TAK1 kinase-dependent inflammation and obesity in male mice. Nat. Commun..

[86] Hoeger B., Nadolni W., Hampe S., Hoelting K., Fraticelli M., Zaborsky N., Madlmayr A., Sperrer V., Fraticelli L., Addington L. (2023). Inactivation of TRPM7 Kinase Targets AKT Signaling and Cyclooxygenase-2 Expression in Human CML Cells. Function.

[87] Sun L., Hua Y., Vergarajauregui S., Diab H.I., Puertollano R. (2015). Novel Role of TRPML2 in the Regulation of the Innate Immune Response. J. Immunol..

[88] Ma S., Wang D.H. (2021). Knockout of Trpa1 Exacerbates Renal Ischemia-Reperfusion Injury with Classical Activation of Macrophages. Am. J. Hypertens..

[89] Bertin S., Aoki-Nonaka Y., Lee J., de Jong P.R., Kim P., Han T., Yu T., To K., Takahashi N., Boland B.S. (2017). The TRPA1 ion channel is expressed in CD4+ T cells and restrains T-cell-mediated colitis through inhibition of TRPV1. Gut.

[90] Sahoo S.S., Majhi R.K., Tiwari A., Acharya T., Kumar P.S., Saha S., Kumar A., Goswami C., Chattopadhyay S. (2019). Transient receptor potential ankyrin1 channel is endogenously expressed in T cells and is involved in immune functions. Biosci. Rep..

[91] Feng J., Yang P., Mack M.R., Dryn D., Luo J., Gong X., Liu S., Oetjen L.K., Zholos A.V., Mei Z. (2017). Sensory TRP channels contribute differentially to skin inflammation and persistent itch. Nat. Commun..

[92] Ueno K., Saika S., Okada Y., Iwanishi H., Suzuki K., Yamada G., Asamura S. (2023). Impaired healing of cutaneous wound in a Trpv1 deficient mouse. Exp. Anim..

[93] Di A., Gao X.P., Qian F., Kawamura T., Han J., Hecquet C., Ye R.D., Vogel S.M., Malik A.B. (2011). The redox-sensitive cation channel TRPM2 modulates phagocyte ROS production and inflammation. Nat. Immunol..

[94] Boukenna M., Rougier J.S., Aghagolzadeh P., Pradervand S., Guichard S., Hämmerli A.F., Pedrazzini T., Abriel H. (2023). Multiomics uncover the proinflammatory role of Trpm4 deletion after myocardial infarction in mice. Am. J. Physiol. Heart Circ. Physiol..

[95] Weber K.S., Hildner K., Murphy K.M., Allen P.M. (2010). Trpm4 differentially regulates Th1 and Th2 function by altering calcium signaling and NFAT localization. J. Immunol..

[96] Schilling T., Miralles F., Eder C. (2014). TRPM7 regulates proliferation and polarisation of macrophages. J. Cell Sci..

[97] Wu Q.Y., Sun M.R., Wu C.L., Li Y., Du J.J., Zeng J.Y., Bi H.L., Sun Y.H. (2015). Activation of calcium-sensing receptor increases TRPC3/6 expression in T lymphocyte in sepsis. Mol. Immunol..

[98] Reese R.M., Dourado M., Anderson K., Warming S., Stark K.L., Balestrini A., Suto E., Lee W., Riol-Blanco L., Shields S.D. (2020). Behavioral characterization of a CRISPR-generated TRPA1 knockout rat in models of pain, itch, and asthma. Sci. Rep..

[99] Nevius E., Srivastava P.K., Basu S. (2012). Oral ingestion of Capsaicin, the pungent component of chili pepper, enhances a discreet population of macrophages and confers protection from autoimmune diabetes. Mucosal Immunol..

[100] Uchida K., Tominaga M. (2014). The role of TRPM2 in pancreatic beta-cells and the development of diabetes. Cell Calcium.

[101] Bousquet J., Czarlewski W., Zuberbier T., Mullol J., Blain H., Cristol J.P., De La Torre R., Pizarro Lozano N., Le Moing V., Bedbrook A. (2021). Potential Interplay between Nrf2, TRPA1, and TRPV1 in Nutrients for the Control of COVID-19. Int. Arch. Allergy Immunol..

[102] Diep P.T. (2022). TRPV1, Nrf2, and COVID-19: Could Oxytocin Have a Beneficial Role to Play?. Int. Arch. Allergy Immunol..

[103] Kuebler W.M., Jordt S.E., Liedtke W.B. (2020). Urgent reconsideration of lung edema as a preventable outcome in COVID-19: Inhibition of TRPV4 represents a promising and feasible approach. Am. J. Physiol. Lung Cell. Mol. Physiol..

